# Resisting Visual, Phonological, and Semantic Interference – Same or Different Processes? A Focused Mini-Review

**DOI:** 10.5334/pb.1184

**Published:** 2023-04-11

**Authors:** Coline Grégoire, Steve Majerus

**Affiliations:** 1 Psychology & Neuroscience of Cognition Research Unit, University of Liège, Belgium; 2 Fund for Scientific Research FNRS, Belgium

**Keywords:** resistance to interference, interference, domain-general, domain-specific, inferior frontal gyrus

## Abstract

The unitary nature of resistance to interference (RI) processes remains a strongly debated question: are they central cognitive processes or are they specific to the stimulus domains on which they operate? This focused mini-review examines behavioral, neuropsychological and neuroimaging evidence for and against domain-general RI processes, by distinguishing visual, verbal phonological and verbal semantic domains. Behavioral studies highlighted overall low associations between RI capacity across domains. Neuropsychological studies mainly report dissociations for RI abilities between the three domains. Neuroimaging studies highlight a left vs. right hemisphere distinction for verbal vs. visual RI, with furthermore distinct neural processes supporting phonological versus semantic RI in the left inferior frontal gyrus. While overall results appear to support the hypothesis of domain-specific RI processes, we discuss a number of methodological caveats that ask for caution in the interpretation of existing studies.

## 1. Introduction

### Resistance to interference: some definitions

Resistance to interference (RI) has been defined as “*the ability to ignore or inhibit irrelevant information while executing a plan*” (p.397) (Dempster & Corkill, 1999). Harnishfeger (1995) made a critical distinction between inhibition and RI by proposing that the latter prevents irrelevant information from entering the mental workspace while the former involves active removal of information no longer useful for the current task. RI was initially defined as a property of memory processes. The Classical Interference Theory (McGeoch, 1932) claimed (McGeoch & Underwood, 1943; Melton & Irwin, 1940; see Demonty et al., 2022 for a recent review on interference and forgetting) that we are less likely to remember and recall an item (item A) if associated with a retrieval cue (item B) that has been paired with another item (item C) during the maintenance period. Item C is considered to interfere here with the maintenance and correct retrieval of item A (Müller & Pilzecker, 1900), a situation illustrating reactive RI. Next, the concept of resistance to proactive interference (Neoclassical Interference Theory; Postman, 1961; Postman & Underwood, 1973; Underwood & Ekstrand, 1966) was introduced to characterize the situation when pre-existent information (so-called ‘extra-experimental sources of interference’) is interfering with novel information to be learned. In 2000, Nigg further distinguished RI from other inhibitory-related processes by proposing that RI prevents competition and/or distraction between stimuli and/or resources in order to maintain a certain level of performance. In 2004, Friedman and Miyake specified three different types of RI: resistance to distractor interference (i.e., to resist interference created by irrelevant stimuli while performing a task), resistance to proactive interference (i.e., prevent intrusions [into memory] by stimuli that were previously relevant but are no longer relevant), and prepotent response inhibition (i.e., the ability to purposely suppress dominant, automatic, or prepotent responses).

### Domain-General and Domain-Specificity of Resistance to Interference

While there has been ample interest in the definition of different, context-dependent types of RI, a fundamental question that has received less explicit consideration is whether these different processes are central processes or whether they are specific to each stimulus domain. In other words, is (proactive, distractor-related, …) resistance to irrelevant auditory-verbal or visual stimuli supported by the same general processes or are these processes specific to the representational properties of each domain, with the further possibility of the existence of domain-general and domain-specific processes at the same time? While RI is often considered to be a central, executive control process (De Baene et al., 2015; Green, 1998; Miyake et al., 2000), some authors have considered that RI may need to be distinguished according to the stimulus domain towards it is applied. Dempster (1993) identified perceptual RI for resisting to auditory or visual stimuli like sounds or symbols and distinguished it from linguistic RI (resistance to relevant linguistic units such as words or sentences) and motor RI (resistance to irrelevant motor acts such as pushing a specific button). In some types of computational models, RI is indeed modelled as a processing property of the representational systems themselves: once a stimulus has been activated/recalled, it is immediately deactivated via algorithms embedded in the representational layers (Oberauer et al., 2012; Schneider, 1993; Schneider & Detweiler, 1988). This makes sense given that interference rather occurs between stimuli from the same domain than between stimuli from different domains, hence within-domain control of interference is a particularly important cognitive process (Oberauer et al., 2012). In contrast, other models of RI and inhibitory-like processes have focused on a hierarchical organization of executive control processes without distinguishing domain specific RI processes (Frank et al., 2001; O’Reilly et al., 2010; Wiecki & Frank, 2012). Furthermore, some models of cognitive control consider the co-existence of domain-specific and domain-general control processes and make a distinction between “primary” and “secondary” controllers. This type of models considers that visual perception, language or motor domains may have their own primary controllers, while secondary (central) controllers operate, moderate, inhibit, and synchronize primary controllers (Verbeke & Verguts, 2021; Verguts, 2017a, 2017b). The question of domain-general and domain-specific RI processes is a central question for the theoretical modelling of RI as the answer to this question will determine whether RI processes should be directly integrated into the processing properties of specific stimulus domains, whether they are better modelled as stimulus-independent, central control mechanisms, or whether both types of situations need to be considered.

### Domain-General or Domain-Specific Interference: A narrative literature review

The aim of this review paper is to examine behavioral, neuropsychological and neuroimaging empirical evidence for and against domain-general RI processes. While distinguishing verbal versus visual domains, we will also distinguish phonological and semantic subdomains within the verbal domain given that there is ample behavioral, neuropsychological and neuroimaging evidence for a separation of these two representational domains (Gorno-Tempini et al., 2011; Patterson et al., 1987). We hypothesize that, if RI are domain-specific, i.e., directly embedded within the representational domains on which they operate, then a separation of RI capacity for visual versus phonological versus semantic information should be observable. Alternatively, if RI processes are (also) domain-general, medium to strong associations for RI capacity across domains should be observable. We reviewed behavioral, neuropsychological and neuroimaging studies in the light of these two main hypotheses. More specifically, for behavioral studies in healthy participants, we targeted studies that used an interindividual differences approach and compared RI across domains while probing the same type of RI (for example, verbal proactive vs. visual proactive). In case of domain-general RI processes, RI performance in one stimulus domain should correlate robustly with RI performance in the other stimulus domain (note that this outcome would not rule out the possibility of *additional* domain-specific RI processes); no or little correlation would be in favor of domain-specific RI processes. Importantly, we were not interested in examining whether there is cross-domain interference or not as this question would not necessarily inform us about the domain-general and/or domain-specific nature of RI processes. On the one hand, it could be argued that if RI processes are domain-specific, cross-domain interference effects should be smaller than within-domain interference effects for comparable tasks and material. However, it can also be argued that interference effects are intrinsically smaller when crossing domains because the stimuli are less similar and hence less prone to interference (Logie et al., 1990; Morey et al., 2013; Shah & Miyake, 1996; Vergauwe et al., 2010). We therefore decided to focus exclusively on studies examining whether the capacity to *resist* interference in one stimulus domain (e.g., visual Stroop task) determines (correlates with) the capacity to *resist* interference in another stimulus domain (e.g., auditory-verbal Stroop task) or not. We will use the same rationale when examining neuropsychological studies, but by focusing on associations versus dissociations of deficits in RI for visual vs. phonological vs. semantic-RI tasks. Regarding neuroimaging studies, we will examine the neural substrates associated with RI in different domains and then focus on studies that have compared RI across domains for comparable tasks and RI types. Finally, note that in order to ensure that we are comparing RI between clearly distinct domains, we will not include studies that compared RI for auditorily versus visually presented verbal material. We consider that presenting words auditorily or visually amounts to processing verbal information in both cases, and hence remaining mostly within the same stimulus domain; this situation would thus not be theoretically informative about the question of domain-specific and/or domain-general RI processes.

We used the following literature search strategy for this focused, narrative review. We first searched in the Medline-PubMed and APA PsychInfo databases studies listed with the following keywords (task-name^1^) AND (visual OR verbal OR (domain-specific) OR (domain-general) OR (modality-general) OR (modality-specific) OR semantic OR phonological) AND (interference AND ((resistance) OR (inhibition) OR (control)))) AND NOT motor), without any language restriction (but the title had to be translated in English). Only peer-reviewed empirical papers were included. Papers were then screened according to the specific search questions and research designs defined in the previous paragraph, and only studies corresponding to the specified research designs were retained. Additional relevant references cited in the examined papers that did not show up in the initial literature search were also included (see Appendix).

## 2. Behavioral studies comparing verbal and visual RI in healthy participants

Many studies have investigated RI in verbal or visual domains, but very few studies have directly compared RI performance across the two domains. Three studies were identified that corresponded to the search criteria. Morey and Mall (2012) investigated RI at the task level, by comparing performance for serial order reconstruction tasks for verbal or spatial stimuli, to be carried out in single-task or dual-task conditions. In the dual-task conditions, either both verbal and spatial stimuli (uncued condition), or only one of the two types of stimuli (cued condition) had to be maintained and recalled. While in the dual uncued conditions, a moderate-size correlation (*r* = .35) was observed between verbal and spatial recall measures, this was not the case (*r* = .18) in the cued conditions. The cued condition is the most informative here for the question of RI capacity given that this condition is not affected by between-stimulus competition for working memory maintenance (which could explain the correlation in the uncued condition) and RI is needed for selectively maintaining the cued visual or verbal stimuli. Oberauer et al. (2004) also investigated RI capacity across domains by using dual-task working memory paradigm. Oberauer and colleagues presented a list of verbal/visual items followed by a list of visual/verbal items in the dual-task condition, and only one type of list in the single task condition. Participants were then asked to recall one of the lists, a situation similar to the cued condition in the study by Morey and Mall. The authors computed different measures of dual-task interference costs (e.g., subtracting the dual-task score from the corresponding single-task score, proportional drop in performance under dual-task conditions relative to single task performance; absolute differences between single- and dual-task performance). Of the eight possible correlations between verbal and visual dual-task cost scores, virtually all correlations were non-significant. The authors concluded that their data provide no support for domain-general RI processes. Finally, Sulpizio et al. (2022) examined verbal semantic RI capacity via a lexical decision task (i.e., word and nonword strings have to be categorized regarding their lexical status, with the words being either neutral words or taboo words, the latter interfering with the lexical decision response) and a semantic Stroop task (i.e., written color-associated words, such as lawn/strawberry/sky/lemon, are presented in a congruent or an incongruent font) and visual RI via a Simon task (i.e., participants are presented with two horizontally aligned colored squares and are asked to categorized them based on their colors; half of the time the color response button is located on the same side as the stimulus and half of the times it is located in the opposite position). The authors observed a small-to-moderate size correlation (*r* = .25) between the verbal RI measures for the lexical decision and the Stroop task, but a non-significant correlation (*r* = values not reported) between RI measures for the Simon and the lexical tasks, as well as between the Stroop and the Simon tasks. These results also support the existence of domain-specific rather than domain-general RI processes.

We should note here that there is a much larger number of studies that have examined the occurrence of between-domain RI effects by comparing the occurrence of dual-task costs for same-domain and between-domain tasks or for same modality and between-response modality tasks, or by comparing the occurrence of phonological versus semantic interference effects (Araneda et al., 2015; Cowan & Barron, 1987; Cowan & Morey, 2007; Donohue et al., 2013; Driver & Baylis, 1993; Elliott et al., 1998, 2014; Guerreiro et al., 2013; Hanauer & Brooks, 2005; Hazeltine & Wifall, 2011; Hirst et al., 2019; Ikeda et al., 2010; Miles et al., 1989; Redding & Gerjets, 1977; Roelofs, 2005; Tipper et al., 1988). However, for the reasons already specified, we did not include these studies in this review as they do not directly compare interindividual differences in RI capacity across domains. Note that for this section, no study comparing RI for phonological vs. semantic subdomains and corresponding to our search criteria was identified.

## 3. Neuropsychological data

### 3.1 RI for verbal vs. visual information in brain-damaged patients

Next, we examine neuropsychological studies that have compared RI abilities between domains, by focusing first on visual vs. verbal domains. These studies mainly involve patients with (a history of) aphasia and associated verbal working memory and control deficits.

Hamilton and Martin (2005) reported the profile of patient ML with a major left frontal lesion (i.e., left frontal and parietal operculum, with atrophy noted in the left temporal operculum and with mild diffuse atrophy) who showed impaired performance for the interference condition of the verbal Stroop task but not for a closely visuo-spatial variant of the Stroop task or for an antisaccade task (see Table 2 for a description of the tasks). In this patient, verbal and visual RI appeared to show a clear between-domain dissociation, even for closely matched RI tasks such as the Stroop task, and despite patient M.L. having no major naming difficulties (Martin & He, 2004; Martin & Lesch, 1996). More recently, Kuzmina and Weekes (2017) investigated RI in verbal and visual domains in a group of 31 patients with aphasia and healthy controls. Participants were administered a visual Flanker task (see Table 2), a cognitive control task (a rule finding task where participants were presented colored dots changing position and had to guess where the next one would appear), a verbal Stroop task for measuring RI, and an auditory-verbal control task (participants had to detect target stimuli within an auditory sequence while ignoring distractors semantically related to the distractor). Overall, the patients were less accurate in the Stroop task (fluent subgroup: z = –2.32; non-fluent subgroup: z = –2.58) and in the auditory-verbal control task (fluent subgroup: *z* = –2.7; non-fluent: z = –4.10) whereas in the general cognitive control task, only the non-fluent subgroup performed worse than controls (*z* = –2.02). Overall, the patients with aphasia showed stronger impairment in the verbal RI tasks compared to the nonverbal tasks, at least for the fluent subgroup.

In sum, the few studies presented here appear to support a dissociation of verbal versus visual RI abilities in brain injured patients. However, the extent to which these results reflect a more general dissociation between verbal vs. visual impairment remains a partially open question given that all dissociations are one-way, with impairment of verbal RI but preservation of visual RI abilities, in patients with associated language impairment.

### 3.2 RI for phonological vs. semantic information in brain-damaged patients

Next, we turn to the neuropsychological studies that have investigated dissociations between RI for phonological vs semantic domains within the verbal domain. These studies also mainly involve patients with aphasia.

Martin and Lesch (1996) presented the language and working memory profiles of three left-hemisphere damaged patients, the patients AB, ML and MS. Patients AB and ML were considered to have greater difficulties for maintaining semantic information in verbal tasks (as evidenced for example by more pronounced difficulties for word than nonword stimuli), a deficit that was subsequently interpreted as reflecting a deficit in resisting semantic interference. Indeed, these patients showed a significant proportion of intrusion errors in WM recall tasks, involving the production of words belonging to previous trials. This type of errors is uncommon for patients with a short-term maintenance deficit, as these patients will generally show increased forgetting instead of presenting increased recall rates for previously presented word. This pattern of results has been interpreted as an overactivation of semantic information and a difficulty of RI stemming from this semantic overactivation (Freedman & Martin, 2001; Martin & He, 2004). Patient M.S., who was instead supposed to present a decay-based, phonological WM impairment indeed did not show increased rates of intrusion errors involving items from earlier trials. This interpretation has been subsequently refined by paradigms designed to measure RI for semantic and phonological information more directly. One of these paradigms is the recent negative task in which lists of words are presented, each list being followed by a probe word for short-term probe recognition (Hamilton & Martin, 2005). Negative probe words are phonologically or semantically related to a word of either the current or a previous memory list. Here, no dissociation between phonological and semantic RI was observed, patient ML, supposed to have a specific semantic RI deficit, being generally slower in both conditions compared to the control group. On the other hand, Barde et al. (2010) demonstrated a distinction between phonological and semantic RI by administering the same type of recent negative task to 20 aphasic patients with left hemisphere lesions and phonological or semantic working-memory (WM) deficits. They showed that patients with a phonological WM deficit showed a stronger RI deficit for phonological negative probes in the recent negative task while patients with a semantic WM deficit showed a stronger deficit for semantic negative probes. More specifically, via stepwise regressions, they observed that a phonological composite WM score explained between 19% and 33% of the phonological interference score; a semantic WM composite explained between 2% and 29% of the semantic interference score. Each time, adding the other WM composite score to the regression did not increase predictive power. At the same time, these dissociations cannot be interpreted in an unambiguous manner given that the greater difficult to reject phonological/semantic distractors could stem from the reduced precision of phonological/semantic representations in WM given the associated, domain-specific WM impairment.

More recently, McCall et al. (2022) investigated phonological and semantic control in 32 aphasic patients with left hemisphere lesions via a switching-control task. Participants had to switch between the selection of 2 or 3 targets that are unrelated or phonological/semantic related, or just select 1 target. Here, interference was calculated by subtracting transformed time per target selection in the unrelated condition from transformed time per target selection in the phonological/semantic related condition. The authors observed impaired performance for the phonological interference condition (*d* = 0.59), but not for the semantic interference condition (*d* = 0.23).^2^ Correlations between the semantic and phonological interference measures were not significant (sequence length 1: *r* = .30; sequence length 2: *r* = .34; sequence length 3: *r* = .25).

Finally, Schnur et al. (2006) used a paradigm involving the progressive build-up of semantic interference, the cyclic naming task. In this task, semantic interference is instaured by having participants repeatedly name the same pictures involving objects from the same or a different semantic category (see Table 1). For same category objects, semantic representations will be progressively over-activated, leading to interference during naming as reflected by increased naming latencies for same-category relative to different-category objects over the different naming cycles. These semantic interference effects have been shown to be increased in patients with aphasia, and this particularly for patients with prefrontal lesions (Biegler et al., 2008; Damian et al., 2001; Jefferies et al., 2007; Schnur et al., 2006, 2009; Thompson et al., 2017). While phonological variants of this paradigm (the pictures to be named refer to phonologically similar names) have also been developed and shown to lead to increased interference effects in patients with aphasia (Hodgson et al., 2005), there are no direct comparisons so far between phonological and semantic interference build-up conditions of this task.

**Table 1:** Description of verbal and visual RI tasks cited in this review.


TASK	SOURCE	TYPE OF RI	STIMULI (EXAMPLES)	DESCRIPTION	MAIN EFFECTS	DOMAINS

Stroop	Stroop (1935), Parris et al. (2022)	Interference caused by conflict between font color and word reading	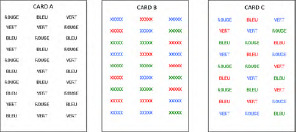	Naming of the color font as quickly as possible	Increased naming latencies in the incongruent condition (CARD C) compared to the congruent condition	Verbal

Verbal Go/No-Go		Interference between target and non-target stimulus status	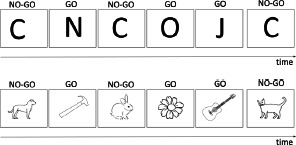	Pressing a key as quickly as possible for a specific word/letter/semantic category (Go condition) and ignore all other stimuli (No-Go condition))	More commission errors and slower response times for No-Go trials	Verbal

Negative-priming task	Tipper (1985), (2001); Tipper & Cranston (1985); Tipper & Driver (1988)	Interference caused by the change of stimulus status: a distractor stimulus becomes a target stimulus	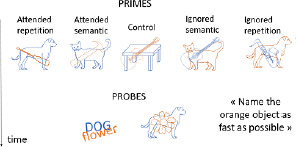	Naming objects in orange font as quickly as possible	Increased reaction times and error rates for a target that had to be ignored in a previous trial	Semantic

Semantic blocked cyclic naming	Schnur et al. (2006); Schnur et al. (2009)	Build-up of semantic interference	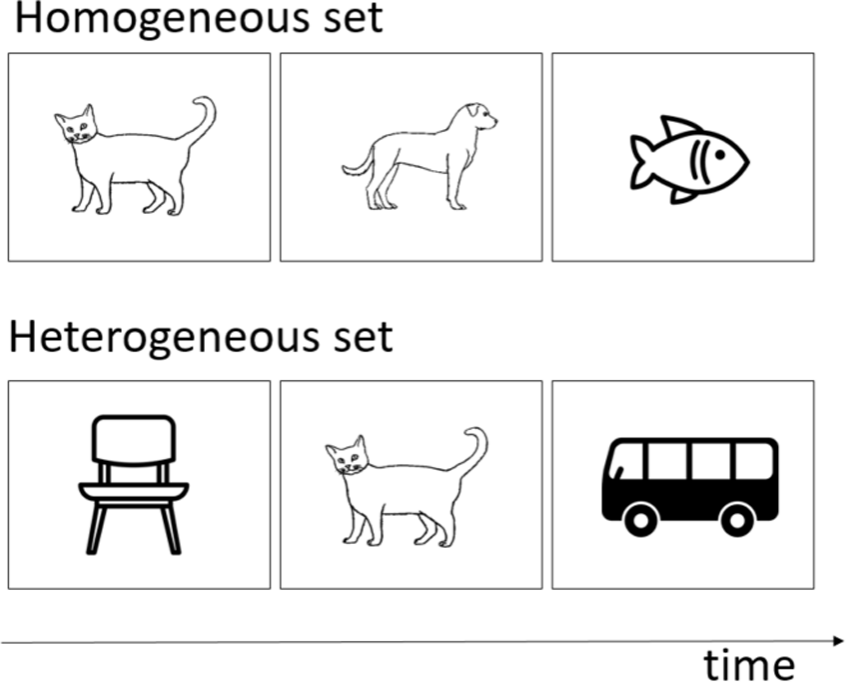	Naming the pictures as quickly as possible	Slower response times and increased error rates when the repeatedly presented objects are from the same semantic category	Semantic

Phonological blocked cyclic naming	Damian, 2003; Hodgson et al., 2005	Build-up of phonological interference	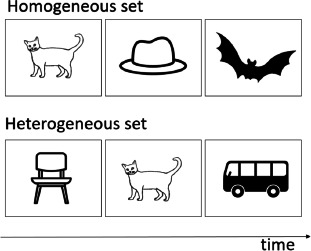	Naming the pictures as quickly as possible	Slower response times and increased error rates when the repeatedly presented objects are phonologically similarly	Phonological

Picture-Word Interference	Glaser & Düngelhoff (1984)	Build-up of phonological and/or semantic interference	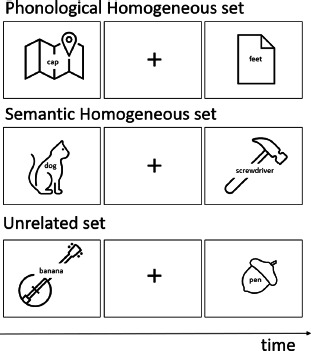	Naming the pictures as quickly and accurately as possible while ignoring the superimposed words	Slower response times and increased error rates for related distractor	Verbal

Similarity-Judgement Task	Attout et al. (2022), Snyder et al. (2007)	Build-up of phonological and/or semantic interference	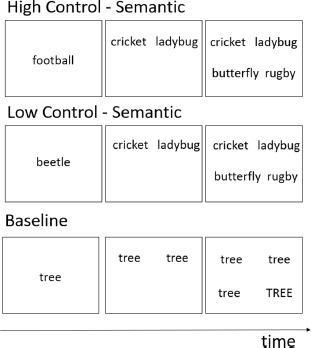	Choosing which word at the bottom of the screen is most similar to the two words at the top of the screen, as quickly and accurately as possible.	Slower response times and increased error rates in the high control conditions as the prime word induced interference.	Verbal

Dual-Interference Task	Pashler (1994)	Build-up of verbal or visual interference	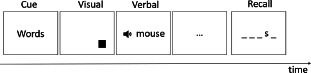	Concurrently and alternatively process two types of information. The tested condition (here, verbal) can be cued or uncued.	Increased error rates and longer reaction time in the tested condition (here, verbal)	Verbal and/or Visual

Flanker task	Eriksen & Eriksen (1974)	Response interference	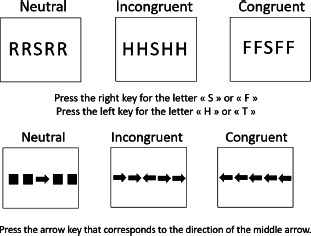	Pressing of a response button according to a predefined response setup. This target can be flanked by a stimulus requiring the same or a different response as the target.	Increased reaction times if target is flanked by an item requiring a different response (interference) and reduced reaction times if target is flanked by an item requiring the same response (facilitation)	Verbal

						Visual

			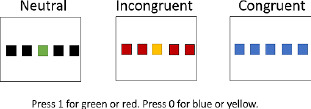			

Visual Go/No-Go		Resist to a visual stimulus	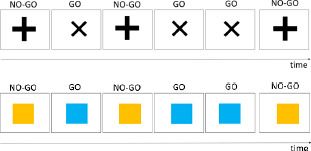	Participants have to press a key as quickly for target symbols (e.g.,Ï), (Go condition), and ignore all other stimuli (No-Go condition).	More commission errors and slower response times for No-Go trials	Visual

Antisaccade	Adapted from Noorani (2014)	Resist to visual stimulus	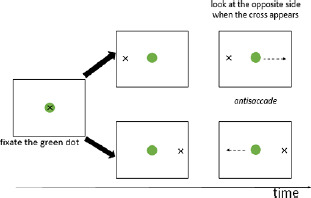	Participants have to fixate a central stimulus and keep fixating the stimulus (green dot). Next a target (the cross) appears to the left, or the right of the dot and participants have to make an eye saccade to the opposite side (=antisaccade).	More errors/slower eye movements for antisaccade trials	Visual


**Table 2:** Summary table of neuroimaging studies of RI in the visual domain.


STUDIES	REGIONS	TASKS

Wager et al. (2005)	bilateral anterior insula/frontal operculum and anterior prefrontal, right DLPFC and premotor, and parietal cortices	Go/No-Go (letters), Flanker task (colors) and a stimulus–response compatibility (arrows) task

Nee et al. (2007)	right DLPFC right DLPFC, right IFG, insula	Flanker task Go/No-Go

McNab et al. (2008)	right IFG	Go/No-Go (squares), Flanker task (arrows) and Stop task (arrows)

Simmonds et al. (2008)	superior medial wall, right prefrontal regions, left premotor cortex, bilateral inferior parietal regions, bilateral occipital regions, bilateral putamen and insula	Meta-analysis on Go/No-Go brain activation

Zhu et al. (2010)	right IFG, MFG, PCG left MFG, PCG	Flanker task (arrows)

Weeks et al. (2020)	right IFG pars triangularis, right MTL, bilateral visual areas	Retrocue recognition task (faces, objects, bodies, and scenes)


*Notes*: DLPFC = dorsolateral prefrontal cortex, IFG = inferior frontal gyrus, MFG = medial frontal gyrus, MPFC = medial prefrontal cortex, PCG = precentral gyrus, MTL = medial temporal lobe, SGF = superior frontal gyrus.

In sum, selective RI deficits for phonological vs. semantic information have been reported, but these dissociations are not systematic and could reflect, at least partly, domain-specific WM impairment rather than domain-specific RI impairment.

## 4. Neuroimaging studies

In this final section, we examine the neuroimaging studies that have examined the neural substrates of RI for visual vs. verbal domains, including the distinction between phonological and semantic verbal domains. We will first focus on neuroimaging studies that have examined visual RI and verbal RI separately. We will then review the few studies that have directly contrasted visual and verbal RI.

### 4.1 The functional neural substrates of visual and verbal RI

One of the first studies focusing more specifically on RI in the visual domain is a study by Wager et al. (2005). The authors explored the neural substrates associated with RI in a Flanker task (see Table 1), a go/no-go task and a stimulus-response compatibility task (see Table 1). Note however that both tasks also have a strong response inhibition component. The authors observed the recruitment of a large bilateral network involving, among other areas, the pars opercularis of the bilateral inferior frontal gyrus (IFG) when resisting to irrelevant visual stimuli. These findings are also in line with a study by McNab et al. (2008) which furthermore aimed at separating RI and working memory/attentional control components among three executive tasks (Go/No-Go, Flanker and a stop task) and two working-memory tasks (one spatial, one verbal). Using conjunction analyses over condition-specific univariate neural activity peaks, the authors observed that the right IFG as supporting more specifically the RI component while parietal cortices were associated with the working memory/attentional control components. In a further fMRI study, increased activity of the medial frontal and precentral gyri and decrease of the right IFG activity were observed during a Flanker task (see Table 1) (Zhu et al., 2010). A meta-analysis by Simmonds et al. (2008) on the visual Go/Go-No task identified the right middle/inferior frontal gyri as well as the bilateral inferior parietal and occipital regions to be specifically associated with the suppression of irrelevant, interfering visual stimuli. Another neuroimaging meta-analysis conducted by Nee et al. (2007) confirmed the implication of the right IFG/dorsolateral prefrontal cortex when interference resolution on visual information is involved. More recently, Weeks et al. (2020) investigated neural representations underlying visual RI by comparing responses to target and non-target stimuli during the delay phase of a WM task in young and older adults. They manipulated face, object, body, and scene stimuli. They observed recruitment of bilateral occipital and medial temporal cortices associated with visual processing. They also observed a higher activation in the right IFG when non-target stimuli occurred and had to be suppressed. However, one limit of this study, in the context of this review, is that the visual material (faces, objects, bodies, scenes) could be easily verbalized.

Regarding verbal RI, one of the first studies that was conducted is a study by Petersen et al. (1999), which actually was one of the first neuroimaging studies on RI more generally. The authors examined the neural substrates associated with the verbal Stroop task (see Table 1) and observed increased activity peaks in the bilateral inferior frontal gyri (IFG) as well as in the anterior cingulate for the RI and response selection stages of this task. A number of subsequent studies have replicated this finding, and this mainly for the left IFG (Gruber et al., 2002; Manard et al., 2017; Parris et al., 2019; Peterson et al., 2002; Taylor et al., 1997; van Veen & Carter, 2005) (see Table 3). Other studies examined the neural substrates associated with the Picture-Word Interference task (i.e., naming pictures as quickly and accurately as possible while ignoring superimposed words; see Table 1). These studies also highlighted the involvement of the bilateral IFG (i.e., orbitomedial prefrontal cortex), together with the left mid middle temporal gyrus, left posterior superior temporal gyrus, and left anterior cingulate cortex (Abel et al., 2009, 2012; de Zubicaray et al., 2001; Gauvin et al., 2021).

**Table 3:** Summary table of neuroimaging studies of RI in the verbal domain.


STUDIES	REGIONS	TASKS

Paulesu et al. (1997)	anterior triangular portion of the left IFG and the left thalamus	Phonemic fluency task Semantic fluency task

	posterior opercular portion of the left IFG for phonemic fluency left retrosplenial region of the left IFG for semantic fluency	

Taylor et al. (1997)	left IFG	Stroop task

Peterson et al. (1999)	bilateral IFG, anterior cingulate	Stroop task

Leung et al. (2000)	anterior cingulate, insula, premotor and IFG	Stroop task

de Zubicaray et al. (2001)	semantic: left mid middle temporal gyrus, left posterior superior temporal gyrus, left anterior cingulate cortex, bilateral orbitomedial prefrontal cortex	Picture-Word Interference Paradigm

Gruber et al. (2002)	anterior cingulate	Stroop task

Peterson et al. (2002)	anterior cingulate, supplementary motor, visual association, inferior temporal, inferior parietal, inferior frontal, and dorsolateral prefrontal cortices sand the caudate nuclei	Simon and Stroop tasks

McDermott et al. (2003)	semantic: left anterior/ventral IFG, approximate, BA47), left posterior/dorsal IFG (BA44/45), left superior/middle temporal cortex (BA22/21), left fusiform gyrus (BA37), and right cerebellum. phonological: left IFG (near BA6/44, posterior to the semantic regions within IFG described) and within bilateral inferior parietal cortex (BA40) and precuneus (BA7)	Blocked-cyclic naming paradigm

van Veen & Carter (2005)	anterior cingulate, prefrontal, and parietal brain regions	Stroop task

Snyder et al. (2007)	phonological inhibitory control for words only: IFG phonological inhibitory control for nonwords: precuneus and supramarginal areas	Similarity-judgment and matching tasks with high and low conflict levels

Abel et al. (2009)	semantic: left orbitofrontal gyrus, left medial middle temporal gyrus, left angular gyrusphonological: left supramarginal gyrus	Picture-Word Interference Paradigm

Abel et al. (2012)	semantic: left middle temporal gyrus, left superior and inferior parietal lobule, and left inferior/middle FG phonological: right middle temporal gyrus, left precuneus, left inferior parietal lobule, left middle temporal and frontal gyri	Picture-Word Interference Paradigm

Manard et al. (2017)	bilaterally in the inferior frontal operculum and insula, and in the left precentral, inferior parietal and superior occipital gyri	Stroop task

Parris et al. (2019)	semantic: left IFG, right mediodorsal thalamus	Stroop task

Klaus & Hartwigsen (2019)	semantic: anterior left IFG phonological: posterior left IFG	Category member vs. rhyme generation task

Attout et al. (2022)	semantic: bilateral angular gyrus, bilateral middle temporal gyrus and bilateral pars opercularis, orbitalis and triangularis phonological: pars triangularis of the bilateral inferior frontal gyrus and to the left middle temporal gyrus.	Similarity-judgment task


*Notes*: IFG = Inferior frontal gyrus.

**Figure 1:**
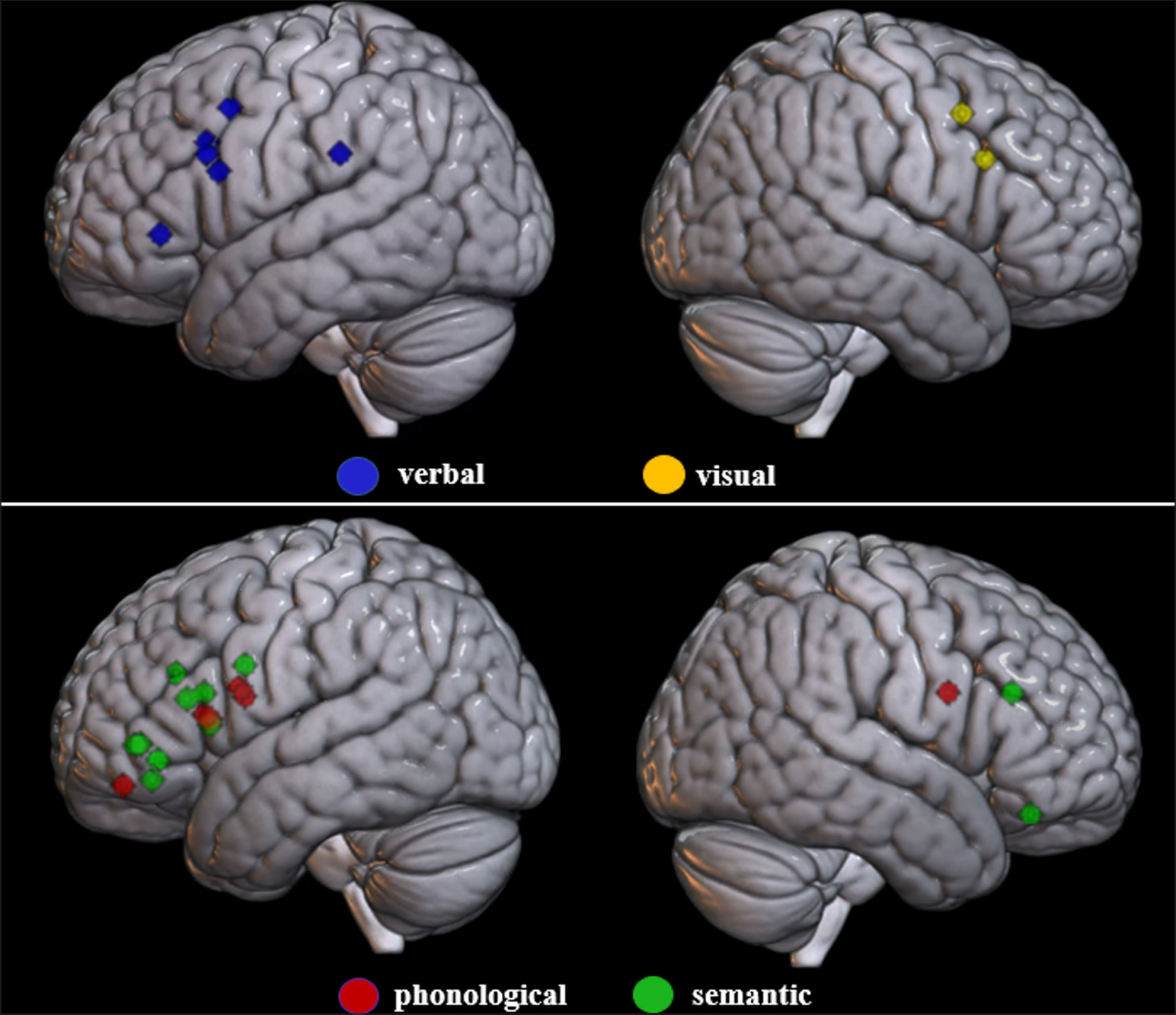
Summary of the activity peaks observed in the left and right frontal lobes for between-domain contrasts of RI. *Notes*: This figure was built by extracting first the MNI coordinates associated with contrasts between verbal semantic, verbal phonological and/or visual RI from the studies reported in this review. The selected MNI coordinates were then assembled using the WFU Pick Atlas (Maldjian et al., 2003, 2004) for each domain and displayed with a sphere shape of 5mm radius. These WFU-generated masks were then overlaid on a 3D render MRI template using MRICroGL (http://www.nitrc.org).

In sum, the different studies reviewed here seem to converge on the involvement of the right IFG in visual RI and the left or the bilateral IFG in verbal RI (see also Figure 1 and Table 3). Other neural regions may also be involved in RI depending on the task used but it is unclear to what extent these regions are associated specifically to RI or to more specific verbal and visual processes.

### 4.2 Direct comparison of neural substrates involved in verbal versus visual RI

Next, we focus on studies that have directly contrasted RI in verbal and visuo(-spatial) tasks (see Table 4). Morimoto et al. (2008) presented two different versions of the Flanker task (Table 2): either a target color word that was flanked by a colored patch (visual RI) or a target-colored patch that was flanked by a color word (verbal RI). The left IFG showed higher activity levels in the verbal RI condition while the right IFG showed higher activity levels in the visual RI condition. These results echo the studies reviewed in the previous sections, indicating a possible left/right hemisphere distinction for verbal vs. visual RI. Schumacher et al. (2011) compared performance for visual and verbal/auditory versions of the Flanker task. Participants were presented two auditory or visual letters (A, B, C or D) and had to respond to the identity of the second letter while ignoring the first. They had to press a right key for A or B, and a left key for C or D. Interference was manipulated by presenting congruent trials involving the same response button for the two successive letters (e.g., B and A) or incongruent trials involving two different response buttons (e.g., B and D). At the behavioral level, the same congruency effect was observed (Eriksen & Eriksen, 1974) for auditory and visual modalities, meaning that the participants were generally slower to perform the incongruent trials rather than the congruent trials. At the neuroimaging level, the authors observed that verbal RI was associated with the left IFG while visual RI was associated with medial prefrontal, occipital and parietal areas as well as the putamen and the thalamus (see Table 4). Some areas were also associated with both verbal and visual RI: the bilateral precentral gyri and left superior frontal gyrus, the supramarginal gyrus, the supplementary motor area, and the putamen. However, the results of this study need to be considered with caution regarding the verbal vs. visual RI contrast given that the visual condition was merely the visual presentation of verbal presentation (letters).

**Table 4:** Summary table neuroimaging studies directly comparing RI in visual and verbal domains.


STUDIES	NEURAL REGIONS		TASKS

	VERBAL	VISUAL	

Stephan et al. (2003)	left anterior cingular cortex, left IFG, fusiform gyrus, lateral extrastriate cortex, ventral premotor cortex and posterior IFG, supplementary motor cortex, bilateral primary visual cortex	anterior and posterior right inferior parietal lobule	Letter and visuospatial decision tasks

Morimoto et al. (2008)	left frontal inferior cortex	right frontal inferior cortex	Flanker tasks

Schumacher et al. (2011)	left inferior frontal gyrus	cingulate gyrus, left precentral gyrus, SMA, inferior temporal gyri, left post central gyrus, fusiform gyri, left superior and inferior parietal lobule, left and right middle and inferior occipital gyrus, left superior occipital gyrus, cerebellum, left putamen and thalamus	Flanker tasks

	both: bilateral precentral gyri and left superior frontal gyrus, supramarginal gyrus, supplementary motor area, and putamen		


*Notes*: IFG = Inferior frontal gyrus, SMA = supplementary motor area.

Finally, Stephan et al. (2003) compared RI in verbal and visual domains by contrasting letter and visuospatial decision conditions. Participants were presented words composed of 4 letters, in which the second or the third letter was printed in red font. In the letter decision condition, verbal RI was manipulated by asking participants to ignore the position of the red letter and to indicate whether the word contained the target letter “A”. In the visuospatial decision task, visual RI was manipulated by asking participants to ignore the language-related properties of the words and to judge whether the red letter was located at left or right relative to the center of the word. The authors observed that verbal RI was associated with the left IFG and anterior cingulate cortex while visual RI was associated with the right anterior cingulate and parietal cortices.

In sum, the studies directly comparing verbal and visual RI consistently highlight a specific involvement of the left IFG for verbal RI, and, but less consistently, a specific involvement of the right IFG in visual RI.

### 4.3 Direct comparison of neural substrates involved in phonological versus semantic RI

Finally, we turn to studies that have directly compared RI for phonological vs. semantic information within the verbal domain. Paulesu et al. (1997) contrasted phonemic and semantic (category) fluency tasks. Although fluency tasks are multi-determined cognitive control tasks, they also involve a RI component given that already produced items interfere with subsequent item retrievals and need to be inhibited. The authors observed increased activity levels in the pars triangularis of the IFG for both tasks, but higher activity in the pars opercularis of the left IFG for the phonological task and higher activity of the left retrosplenial cortex for the semantic task. Other studies claimed that the anterior part of the left IFG would support RI and associated cognitive control in semantic tasks while the posterior part would support RI in phonological tasks (Klaus & Hartwigsen, 2019; McDermott et al., 2003). However, Snyder et al. (2007) investigated resistance to semantic and phonological interference using similarity-judgment and matching tasks with high and low conflict levels (see Table 1 for example). The authors observed no significant differences in neural responses in the left IFG between phonological and semantic conditions while showing at the same time generally enhanced activity levels in the left IFG in the high-conflict conditions. Using a similarity-judgment-task (see Table 1) and contrasting also high and low conflict conditions, Attout et al. (2022) recently compared semantic and phonological RI using both univariate and multivariate neuroimaging methods. The authors observed common involvement of the pars triangularis of the bilateral IFG and as well as the left middle temporal gyrus for both phonological and semantic RI, with further more widespread fronto-parietal involvement for semantic RI (see Table 2). Critically, multivariate neural patterns associated with phonological RI in different IFG areas could not predict neural patterns associated with semantic RI in the same areas, and vice-versa. These data indicate that even if similar neural regions may support both phonological and semantic RI, the neural processes involved differ. Finally, studies focusing on the picture-word interference task (see Table 1) also showed an involvement of the left IFG as well as of temporo-parietal cortices in verbal RI (Abel et al., 2009, 2012). Importantly, differences were also observed for phonological versus semantic distractors, with phonological RI being associated with the left supramarginal gyrus and semantic RI with the left orbitofrontal gyrus, left medial middle temporal gyrus and left angular gyrus.

In sum, the neuroimaging studies reviewed here show that the left IFG, supports both phonological and semantic RI, with no clear distinction between anterior and posterior parts for the IFG (see Figure 1). But at the same time, there is also consistent evidence for a neural separation of phonological and semantic RI, semantic RI involving also more posterior temporo-occipital and temporo-parietal cortices. Most importantly, even if the same left IFG regions appear to be involved in both phonological and semantic RI, the specific neural processes supported by these regions appear to differ.

## 5. Discussion and conclusion

This focused mini-review examined behavioral, neuropsychological, and neuroimaging evidence for and against domain-general RI processes. Behavioral studies highlighted overall low associations between RI capacity across visual, verbal phonological and verbal semantic domains. Neuropsychological studies mainly showed dissociations for RI abilities between the three domains. Neuroimaging studies highlighted a left vs. right hemisphere distinction for verbal vs. visual RI, with furthermore distinct neural processes supporting phonological versus semantic RI in the left IFG. Overall, the results appear to support the view of domain-specific rather than domain-general processes. Noteworthy, even if evidence tends to support distinct RI mechanisms, it does not exclude the possible existence of additional, higher level and domain-general control processes over the different domain-specific RI mechanisms (as discussed below). Indeed, there are a number of methodological caveats that need to be discussed and that do not allow to disconfirm the hypothesis of additional, domain-general RI processes.

A first limitation is the small number of studies that have directly addressed the question of domain-specific and/or domain-general RI processes, particularly for behavioral and neuropsychological study designs. At the behavioral level, the vast majority of studies has tried to determine whether there are cross-domain interference effects when using dual task designs, and whether these effects are stronger of not for dual tasks from different versus the same domain. As already noted, this type of studies will inform us about the potential for interference between stimuli/tasks from different domains but not necessarily about the domain-specific and/or domain-general nature of RI processes. For example, strong between-domain dual task costs may reflect increased domain-general attentional control and division demands rather than evidence for domain RI processes. Studies correlating RI measures derived from separate tasks will be more informative as they will allow to directly compare RI ability across domains without the confound of additional executive costs associated with dual tasks. These correlational studies in healthy adults are however rare. At the neuropsychological level, most studies having compared RI across domains are single case studies, revealing only unidirectional dissociations (impaired verbal RI, preserved visual RI). Stronger evidence associated with double dissociations is still lacking and the more general verbal or working memory impairment shown by the patients could also have contributed to the simple dissociations that were observed.

A second cautionary note needs to be raised regarding the comparability of tasks administered for assessing the RI across different domains. While many reported studies used tasks that were very closely matched across domains, with tasks having the same structure and only the nature of the stimuli being changed (e.g., recent negative task using either phonological or semantic probes, Hamilton & Martin, 2005; flanker task with written words vs. coloured patches, Morimoto et al., 2008; similarity-judgment task for words vs nonwords, Attout et al., 2022), this was not always the case (e.g., verbal Stroop task vs. visual Flanker task, Kuzima & Weekes, 2007). Tasks may differ in several other aspects associated with RI, such as the ease of prediction, selection and/or suppression of responses. Hence, we cannot rule out the possibility that the result of lack of association for cross-domain RI comparisons reported in some of the reviewed studies could have been inflated by structural task differences. At the same time, note that cross-domain RI dissociations were also observed in studies that used closely matched task designs. We should however mention here that the use of structurally equivalent tasks across domains does not necessarily guarantee that the amount of interference build-up is exactly the same in the different versions of the task. For example, while Attout et al. (2022) used very strictly matched task designs for probing phonological and semantic RI, by inducing the pre-activation of a phonological or a semantic representation that will interfere with the target response, it will be difficult to determine whether the pre-activations were exactly of the same strength within each domain and interfered to the same extent with the targets. For example, while Attout et al. (2022) observed significant behavioral interference effects for both the phonological and semantic variants of the task, they also observed an interaction with slightly stronger interference effects in the semantic task. In contrast, other design choices could have biased results in favor of an absence of domain-specific RI, specifically when using words for probing both phonological and semantic RI (Hamilton & Martin, 2005, 2007; Martin & Lesch, 1996). Word stimuli may incidentally elicit semantic/phonological processes even when the task focuses on phonological/semantic judgments or when no explicit processing of the word stimuli is required. Jedidi et al. (2021) showed robust involvement of semantic processing areas in temporal cortices during word presentation even when attention was absorbed by a primary visual search task; critically, they even observed the involvement of inferior frontal and mid-temporal areas associated with semantic control and inhibitory processes during incidental, passive processing of word stimuli.

Overall, despite the methodological caveats, the different studies reviewed here appear to favour the existence of domain-specific RI processes rather than purely domain-general RI processes. This does however not mean that RI processes arise exclusively from processes embedded in domain-specific representational systems. This review does not discard the possibility of the co-existence of domain-specific and domain-general, or at the least, central RI control processes, as explicitly or implicitly assumed by some models of cognitive control (Verbeke & Verguts, 2021; Verguts, 2017). The neuroimaging data reviewed here are of particular interest as they suggest, on the one hand, a general implication of prefrontal cortices in RI across domains, but at the same time a specialization of prefrontal cortices for RI as a function of visual vs. phonological vs. semantic stimulus domain. It could be assumed that prefrontal cortices are a central controller of RI by keeping track of task-relevant information, but this can only work in synergy with information-specific representational domains in which task-relevant information is processed. Verbeke and Verguts (2021) proposed a computational model assuming that the synchronization of neural oscillations between prefrontal control systems and posterior domain-specific representational domains allows privileged processing of target vs. non-target information, a situation equivalent to RI (Verbeke & Verguts, 2021). These predictions resonate with the findings of Attout et al. (2022) showing that the same prefrontal regions can be involved in RI for phonological and semantic information, but that they represent different information according to phonological vs. semantic RI situations. By transposing these results to the model by Verbeke and Verguts, it could be argued that the neural state of the prefrontal controller will necessarily differ depending on the type of target information it needs to keep track and type of representational neural network it needs to interact with. Dissociations of RI between verbal and visual domains could occur due to differences in neural connectivity and synchronization between prefrontal control and specific posterior representational systems.

To conclude, the studies reviewed here support a domain-specific rather than a domain-general view of RI processes. However, evidence is still fragmentary and does not allow to rule out domain-general RI processes. Recent computational models of cognitive control are compatible with a hybrid view in which domain-general RI mechanisms can materialize as domain-specific abilities due to the interaction between domain-general RI mechanisms and domain-specific representational systems. But in that case, evidence for the domain-general RI mechanisms should also be observable because a general weakness of the domain-general controller should lead to similar RI impairment across domains. Furthermore, it is important to note that RI is a complex cognitive capacity that likely involves multiple mechanisms and processes, some of which are domain-specific, without excluding the existence of additional domain-general RI control processes. Future studies, comparing RI for different stimulus domains but with structurally and functionally equivalent tasks, are necessary to further elucidate the complex question of domain-general and/or domain-specific RI processes.

## Figures and Tables

**Figure:**
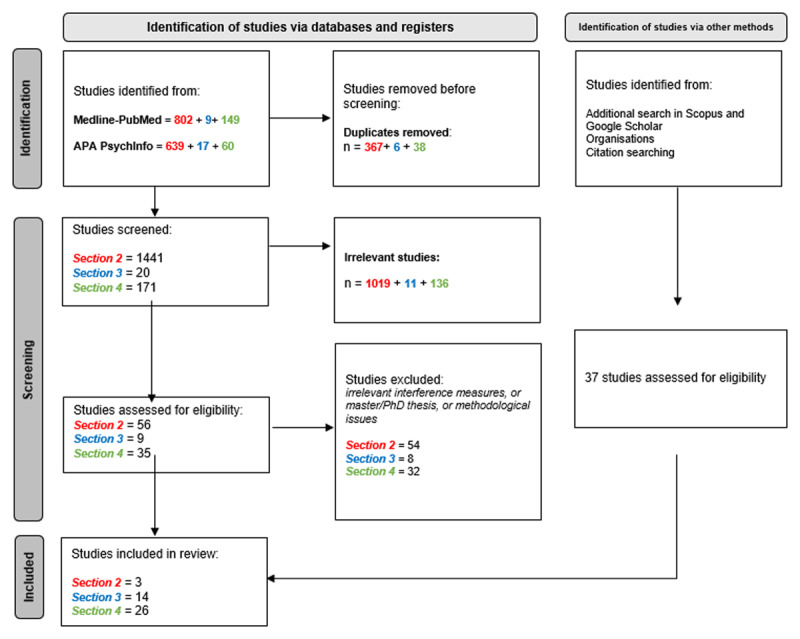
Number of Articles Returned at Every Step of the Research Strategy. *Notes*: also available on https://osf.io/wbk8f/.

## Footnotes

1As task-names, we introduced the names of very common tasks measuring RI or RI-related processes such as “Stroop”, “Flanker”, “Go/No-Go” task.

2Effect size estimated based on test statistics provided in the manuscript (Lenhard, W. & Lenhard, A. (2016). Computation of effect sizes. Retrieved from: https://www.psychometrica.de/effect_size.html. Psychometrica. DOI: 10.13140/RG.2.2.17823.92329).
